# Explicit calculation method for cell alignment in non-circular geometries

**DOI:** 10.1098/rsos.211663

**Published:** 2022-01-19

**Authors:** Hiroki Miyazako, Takaaki Nara

**Affiliations:** Department of Information Physics and Computing, Graduate School of Information Science and Technology, The University of Tokyo, 7-3-1, Hongo, Bunkyo-ku, Tokyo, Japan

**Keywords:** cell alignment, nematic liquid crystals, complex analysis, conformal mapping

## Abstract

The alignment of spindle-shaped cells in two-dimensional geometries induces singular points called topological defects, at which the alignment angle of the cell cannot be defined. To control defects related to biological roles such as cell apoptosis, calculation methods for predicting the defect positions are required. This study proposes an explicit calculation method for predicting cell alignment and defect positions in non-circular geometries. First, a complex potential is introduced to describe the alignment angles of cells, which is used to derive an explicit formula for cell alignment in a unit disc. Then, the derived formula for the unit disc is extended to the case for non-circular geometries using a numerical conformal mapping. Finally, the complex potential allows a calculation of the Frank elastic energy, which can be minimized with respect to the defect positions to predict their equilibrium state in the geometry. The proposed calculation method is used to demonstrate a numerical prediction of multiple defects in circular and non-circular geometries, which are consistent with previous experimental results.

## 1. Introduction

Spindle- and rod-shaped living materials exhibit active behaviours as nematic liquid crystals, known as active nematics [1,2]. Many active nematic systems have been studied, including micro- and nano-biosystems such as microtubule-kinesin compounds [3,4], bacterial colonies [5–8], cell monolayers [9–12], macroscopic tissues [13,14] and animals [15]. In these active nematic systems, the alignment of the spindle-shaped materials induces singular points at which the alignment angle cannot be defined. These singular points are called topological defects. The non-uniformity of the alignment around the defects can cause mechanical imbalances among the components, which may play key roles in the active movement of living matter. Therefore, many experimental and theoretical studies of active nematic systems have focused on the characteristics of the topological defects.

Recent studies have revealed that topological defects in spindle-shaped adhesive cells have different properties from other active nematic systems. Cells cultured in small domains tend to align along the boundaries of the domains. The cell alignment along the boundaries then forces the surrounding cells to align in the same direction at a scale of several hundred micrometres [16,17]. A recent study has shown that such cell alignment leads to the stable and reproducible generation of two topological defects at specific positions when the cells are cultured in a small disc [9]. The defect positions in the disc can be theoretically calculated using nematic liquid crystal theory [9]. It has also been shown that topological defects in certain cell populations can induce apoptosis, which results in extrusion of the cells [10]. Therefore, topological defects are expected to be a mechanical factor for cell function in the field of mechanobiology [18]. Moreover, new materials have been proposed for controlling the alignment and defects in biological nematic cells [19–21]. To systematically control biological functions by topological defects, it is necessary to develop a numerical calculation and design method for predicting and controlling topological defects in cell populations.

There are two approaches for calculating alignment angles of nematic systems in a domain where several topological defects exist. One uses a finite-element method (FEM) [22,23]. A recent study [24] calculated cell alignment and polarization by expressing a contraction force with nematic order parameters. The other method is to derive an explicit expression of the alignment angles in terms of the defect positions. Duclos *et al.* [9] derived a Fourier series expression for the alignment angles of spindle-shaped cells in a disc where two defects exist equidistant from the centre. They determined that there is a certain defect position which minimizes the Frank elastic energy by differentiating the energy with respect to a variable related to the defect positions. The predicted position was consistent with cell culture experiments. Therefore, explicit expressions of the cell alignment can reveal the spatial dependence of the defect positions on the total elastic energy. However, there are some limitations in the explicit expression derived in the previous study; it is based on a Fourier series expansion on a disc and cannot be applied to non-circular geometries. In addition, they considered only a special case when there exist two, symmetrically spaced defects in a disc. To predict defect positions in non-circular geometries, it is necessary to derive general expressions for the cell alignment in non-circular geometries, such as the star-shaped geometry reported in Saw *et al.* [10]

In this paper, we propose an explicit method for calculating cell alignment in non-circular geometries. The proposed method employs a complex-analysis-based approach to express the cell alignment, which enables (i) a simpler expression compared with a Fourier series expression, (ii) generalization of the geometries using conformal mappings, and (iii) easy computation of the elastic energy using contour integration. This paper is organized as follows. In §2.1, we review a physical model and a Fourier series solution for the alignment angles of spindle-shaped cells in a disc according to Duclos *et al.* [9]. Then, we derive an explicit expression for the cell alignment in a unit disc using complex potentials in §2.2. In §3, we combine the derived explicit expression with conformal mapping techniques to compute the cell alignment in non-circular geometries. In §4, we propose a numerical minimization method for the elastic energy by calculating the contour integrals and taking a derivative of the elastic energy with respect to the defect positions. In §5, the proposed method is confirmed by several numerical simulations. Finally, we compare the simulation results with previous experimental results.

In this paper, we use the following mathematical notation. Z and R are the sets of integers and real numbers, respectively. C denotes the complex plane with coordinate *z* = *x* + i*y* (x,y∈R). D : ={z∈C | |z|<1} is the open unit disc in C, and its boundary is defined as $\partial D\;:\;=\{e^{i\theta }\;|\;\theta \in R\}$. We denote the closure of D as $\bar{D}$. For a certain domain $S\subset R^{2}$ whose boundary ∂*S* is a Jordan curve, we denote unit tangential and normal vectors at the boundary as $\hat{s}$ and $\hat{n}$, respectively. For a scalar-valued function *ϕ*(*x*, *y*) on the domain *S*, we define tangential and normal derivatives of *ϕ* at the boundary as $\partial \phi /\partial s\;:\;=(\nabla \phi )\cdot \hat{s}$ and $\partial \phi /\partial n\;:\;=\nabla \phi \cdot \hat{n}$, respectively.

## 2. Explicit expression of cell alignment in a unit disc

### 2.1. Physical model and Fourier series solution of cell alignment in a disc

In this section, we review a physical model and a Fourier series solution of the cell alignment in a disc according to Duclos *et al.* [9]. We model the spindle-shaped cells as rod-like particles with negligible size, aligned along a specific axis denoted by the unit vector ***n***_d_ = (cos*ϕ*(*x*, *y*), sin*ϕ*(*x*, *y*))^T^, where *ϕ*(*x*, *y*) is the alignment angle of a rod, which varies continuously with respect to the position (*x*, *y*). The vector ***n***_d_ is called the ‘director’ [25]. We also assume that ***n***_d_ and −***n***_d_ are indistinguishable; that is, ϕ(x,y)+mπ (m∈Z∖{0}) is identified with *ϕ*(*x*, *y*). We define a restricted value of *ϕ*(*x*, *y*) for [ − *π*/2, + *π*/2) as follows:

2.1 $$\;\hat{\;\phi }(x,y)=\phi (x,y)-\pi \left\lfloor \frac{\phi (x,y)+\pi /2}{\pi }\right\rfloor ,$$

where ⌊x⌋=max{m∈Z | m≤x} is a floor function defined in R. Although both *ϕ*(*x*, *y*) and $\hat{\phi }(x,y)$ express the same direction, the two functions are distinguished in our paper.

Let us consider a special case where the cells exist in a disc $D_{R}\;:\;=\{(x,y)\in R^{2}\;|\;x^{2}+y^{2}<R^{2}\}$. On the boundary ∂*D*_*R*_, the cells are forced to align along the disc. That is, the following boundary condition is assumed:

2.2 $$\;\hat{\;\phi }(R\cos\theta ,R\sin\theta )=\theta -\frac{\pi }{2},$$

where *θ* denotes the polar angle on the boundary.

To derive the governing equation of the angle *ϕ*(*x*, *y*), Duclos *et al.* assumed the physical condition called the ‘one constant approximation’ [25]. Then, for a subdomain *S* ⊂ *D*_*R*_ that does not contain singular points corresponding to topological defects of the cells, the total elastic energy (i.e. Frank elastic energy) *F* is expressed as follows:

2.3 $$F=\frac{K}{2}\iint_{S}|\nabla \phi (x,y){|}^{2}\;dx\;dy,$$

where *K* is the Frank elastic constant. By taking the variation of *F* with respect to *ϕ*(*x*, *y*), it is easily found that the angle *ϕ*(*x*, *y*) should satisfy the Laplace equation

2.4 $$\nabla^{2}\phi (x,y)=0\;((x,y)\in S).$$

Therefore, *ϕ*(*x*, *y*) is a harmonic function.

For a precise definition of topological defects, Duclos *et al.* introduced another scalar function *ψ*(*x*, *y*) which satisfies $\nabla \phi (x,y)=\nabla \times (\psi (x,y)e_{z})$, where ***e***_*z*_ = [0, 0, 1]^T^. It is noted that this constraint is equivalent to the Cauchy–Riemann equations and thus *ψ*(*x*, *y*) is the conjugate harmonic function of *ϕ*(*x*, *y*). Using *ψ*(*x*, *y*), a singular point is defined as a point $\tilde{r}$ that satisfies the following Poisson equation:

2.5 $$\nabla^{2}\psi (x,y)=-\pi \delta (r-\tilde{r}).$$

Let $\tilde{S}\subset D_{R}$ be a bounded domain that contains a single singular point $\tilde{r}$; it then holds from Stokes’ theorem and equation (2.5) that

2.6 $$\begin{array}{rl} \int_{\partial \tilde{S}}\nabla \phi \cdot ds & =\int_{\partial \tilde{S}}(\nabla \times \psi (x,y)e_{z})\cdot \;ds\\ & =\iint_{\tilde{S}}(\nabla \times (\nabla \times \psi (x,y)e_{z}))\cdot e_{z}\;dx\;dy\\ & =\iint_{\tilde{S}}(-\nabla^{2}(\psi (x,y)e_{z}))\cdot e_{z}\;dx\;dy\\ & =\pi , \end{array}$$

where *d****s*** is a line element along $\partial \tilde{S}$. This equation implies the angle of a cell changes by +*π* around the singular point in the counter-clockwise direction. We call this singular point a topological defect at $\tilde{r}$ with a topological charge of +1/2 (or a +1/2 defect). More generally, we define a topological defect at $\tilde{r}$ with a topological charge of a half-integer *q* = *m*/2 (m∈Z∖{0}) as a singular point which satisfies the following Poisson equation:

2.7 $$\nabla^{2}\psi (x,y)=-2q\pi \delta (r-\tilde{r}).$$

In the same way as in equation (2.6), we can show that the angle changes by 2*qπ* around the defect with a topological charge of *q*.

In the study of Duclos *et al.*, it was found experimentally that only two +1/2 defects existed when spindle-shaped cells were cultured in a disc *D*_*R*_. To obtain the distribution of the angles of the cells in the disc, they assumed that the two +1/2 defects were placed at the same distance *r*_0_ from the centre of the disc and considered the following Poisson equation:

2.8 $$\nabla^{2}\psi (x,y)=-\pi \{\delta (r-r_{1})+\delta (r-r_{2})\},$$

where

2.9 $$\begin{array}{cc} & r_{1}=\left(r_{0}\cos\left(\frac{\theta_{0}}{2}\right),\;r_{0}\sin\left(\frac{\theta_{0}}{2}\right)\right),\\ & r_{2}=\left(r_{0}\cos\left(-\frac{\theta_{0}}{2}\right),\;r_{0}\sin\left(-\frac{\theta_{0}}{2}\right)\right). \end{array}$$

Note that it follows from the boundary condition (2.2) and the Cauchy–Riemann relation ∂*ϕ*/∂*s* = −∂*ψ*/∂*n* that ∂*ψ*/∂*n* = −1 on the boundary ∂*D*_*R*_. Under this boundary condition for *ψ*, they solved the Poisson equation (2.8) using the Fourier series of *ψ*. Considering the Cauchy–Riemann relations between *ϕ* and *ψ*, the following closed form of *ϕ* was obtained.

2.10 ϕ(r,θ)=−π2−Re(i2log⁡((r−r0 ei(θ+θ0/2))(r−r0 ei(θ−θ0/2))(R2−r0r ei(θ+θ0/2))(R2−r0r ei(θ−θ0/2))R4r02)).

### 2.2. Explicit expression of cell alignment using complex potentials

As described in §2.1, the previous study [9] modelled the cell alignment as a nematic liquid crystal in a disc and derived the closed form of *ϕ* for a special case when there exist two, symmetrically placed, topological defects. In this subsection, we give a simpler and more general expression of the alignment angle *ϕ*(*x*, *y*) for arbitrary positions and number of defects in the unit disc. To this end, we re-formulate the problem described in §2.1 using complex potentials.

We assume that *N* topological defects exist at *z*_*k*_ = *x*_*k*_ + i*y*_*k*_, where |*z*_*k*_| < 1, for *k* = 1, …, *N*. We denote the topological charge of the *k*th defect by *q*_*k*_ for *k* = 1, …, *N* and the set of defect positions by $Z=\{z_{1},z_{2},\ldots ,z_{N}\}$. Let $\hat{\phi }(x,y)$ be the restricted value of *ϕ*(*x*, *y*) to [ − *π*/2, + *π*/2) calculated by equation (2.1). Considering equations (2.2), (2.4) and (2.6), the alignment angle of the cell *ϕ*(*x*, *y*) satisfies the following three conditions:

*ϕ*(*x*, *y*) is a harmonic function in D∖Z.
For any simply connected, bounded domain *D*_*k*_ ⊂ *D* which contains only the *k*th defect, *ϕ*(*x*, *y*) satisfies
  2.11 $$\oint_{\partial D_{k}}\frac{\partial \phi }{\partial s}\;ds=2\pi q_{k}.$$
For the boundary ∂D, $\hat{\phi }(x,y)$ is equal to its tangential angle. That is,
  2.12 $$\;\hat{\;\phi }(\cos\theta ,\sin\theta )=\theta -\frac{\pi }{2}.$$

The following proposition provides an explicit expression of *ϕ*(*x*, *y*) which satisfies the above three conditions.

**Proposition 2.1.**

*For a holomorphic function in* D∖Z *given by*

2.13 $$f_{D}(z\;|\;Z)=-\sum_{k=1}^{N}iq_{k}(\log(z-z_{k})+\log(1-{\bar{z}}_{k}z))-\frac{\pi }{2},$$

*where*

2.14 $$\sum_{k=1}^{N}q_{k}=1,$$

*its real part*, ϕD(x,y | Z)≡Re[fD(z | Z)], *satisfies conditions* (i)–(iii).

**Proof.**

Condition (i) is clearly satisfied because log (*z* − *z*_*k*_) and $\log(1-{\bar{z}}_{k}z)$ are holomorphic on D∖Z and D, respectively, and hence their real parts are harmonic.

For condition (ii), for the *k*th domain *D*_*k*_, since $\arg(1-{\bar{z}}_{l}z)$ is harmonic inside the domain *D*_*k*_ for *l* = 1, …, *N*, we have from Green’s theorem

2.15 $$\int_{\partial D_{k}}\frac{\partial \arg(1-{\bar{z}}_{l}z)}{\partial s}\;ds=\iint_{D_{k}}\nabla^{2}\;\arg(1-{\bar{z}}_{l}z)\;dx\;dy=0.$$

In the same way as described above, $\int_{\partial D_{k}}(\partial \;\arg(z-z_{l})/\partial s)\;ds=0$ when *l* ≠ *k*, because arg(*z* − *z*_*l*_) is harmonic inside *D*_*k*_. Thus, we need to consider only the term $\int_{D_{k}}(\partial \;\arg(z-z_{k})/\partial s)\;ds$. Let *D*_*k*_(*ε*) : = {*z*_*k*_ + *r* e^i*θ*^ | 0 ≤ *r* ≤ *ε*, 0 ≤ *θ* < 2*π*}, where *ε* is a small positive number. Since arg(*z* − *z*_*k*_) is harmonic on $D_{k}\setminus D_{k}(\epsilon )$, it holds from Green’s theorem that

2.16 $$\begin{array}{rl} & \int_{\partial D_{k}}\frac{\partial \;\arg(z-z_{k})}{\partial s}\;ds-\int_{\partial D_{k}(\epsilon )}\frac{\partial \;\arg(z-z_{k})}{\partial s}\;ds\\ & \;=\iint_{D_{k}\setminus D_{k}(\epsilon )}\nabla^{2}\;\arg(z-z_{k})\;dx\;dy=0. \end{array}$$

Then, it holds that

2.17 $$\int_{\partial D_{k}}\frac{\partial \;\arg(z-z_{k})}{\partial s}\;ds=\int_{\partial D_{k}(\epsilon )}\frac{\partial \;\arg(z-z_{k})}{\partial s}\;ds=\int_{0}^{2\pi }\frac{\partial \;\arg(\epsilon \;e^{i\theta })}{\epsilon \partial \theta }\epsilon \;d\theta =2\pi .$$

From the above discussion, we have

2.18 $$\int_{\partial D_{k}}\frac{\partial \phi_{D}}{\partial s}\;ds=\sum_{l=1}^{N}q_{l}\int_{D_{k}}\left(\frac{\partial \;\arg(z-z_{l})}{\partial s}+\frac{\partial \;\arg(1-{\bar{z}}_{l}z)}{\partial s}\right)=2\pi q_{k}.$$

Hence condition (ii) holds for $f_{D}(z\;|\;Z)$.

For condition (iii), on the boundary $z=e^{i\theta }\in \partial D$, $\log(1-{\bar{z}}_{k}\;e^{i\theta })$ is equal to $\log(e^{i\theta })+\log(e^{-i\theta }-{\bar{z}}_{k})$ up to a constant 2*Lπi*, where *L* is an integer. Therefore, the real part of the sum of the pair of $-iq_{k}\log(z-z_{k})$ and $-iq_{k}\log(1-{\bar{z}}_{k}z)$ becomes

2.19 Re[−iqklog⁡(z−zk)−iqklog⁡(1−z¯kz)]=Re[−iqklog⁡(eiθ−zk)]+Re[−iqk(log⁡(eiθ)+log⁡(e−iθ−z¯k)+2Lπi)]=qk arg(eiθ−zk)+qkθ+qk arg(e−iθ−z¯k)−2qkLπ=qk(arg(eiθ−zk)+arg(eiθ−zk¯))+qkθ−2qkLπ=qkθ−2qkLπ.

Since *q*_*k*_ is a half-integer, the restriction of equation (2.19) to [ − *π*/2, *π*/2) by (2.1) becomes *q*_*k*_*θ*. Using equation (2.14), the restricted value ${\hat{\phi }}_{D}(x,y)$ satisfies condition (iii). ▪

**Remark 2.2.**

The condition $\sum_{k=1}^{N}q_{k}=1$ in proposition 2.1 is necessary for the proof of condition (iii). Conversely, we can show $\sum_{k=1}^{N}q_{k}$ is equal to 1 when $f_{D}$ satisfies conditions (i)–(iii) as follows. The total change of $\phi_{D}(x,y)$ along the boundary $w=\int_{\partial D}(\partial \phi_{D}(x,y)/\partial s)\;ds$ should be equal to 2*π* from condition (iii). However, because $\phi_{D}(x,y)$ is harmonic in $D\setminus (\overset{N}{\underset{k=1}{⋃}}D_{k}(\epsilon ))$, the line integral $\int_{\partial D}(\partial \phi_{D}(x,y)/\partial s)\;ds$ is equal to $\sum_{k=1}^{N}\int_{\partial D_{k}(\epsilon )}(\partial \phi_{D}(x,y)/\partial s)\;ds=\sum_{k=1}^{N}2\pi q_{k}$ from condition (ii) and Green’s theorem. Therefore, $\sum_{k=1}^{N}q_{k}$ should be equal to 1.

Our expression for $\phi_{D}$ in proposition 2.1 is equivalent to equation (2.10) derived in the previous study [9], when *N* = 2, *q*_1_ = *q*_2_ = 1/2, and the defect positions are given by equation (2.9). Moreover, the imaginary part of $f_{D}(z\;|\;Z)$, denoted by ψD(x,y)≡Im[fD(z | Z)], for that case satisfies the Poisson equation (2.8) because −(1/2*π*)log|*z*| is a Green function of the two-dimensional Laplace operator. Therefore, our result is a general expression for the angle *ϕ*(*x*, *y*) as compared with equation (2.10) without any assumptions on the defect number and positions in the unit disc.

**Figure 1.**
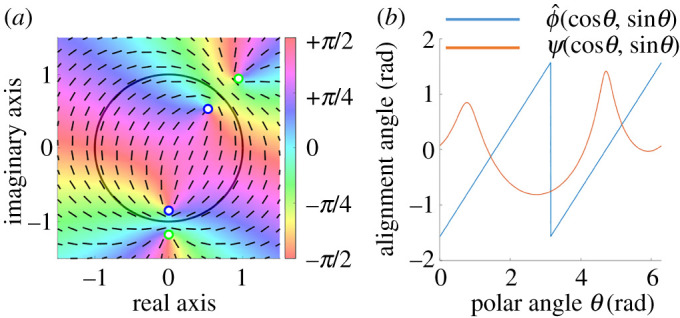
Calculation of cell alignment when two +1/2 defects exist in the unit disc. (*a*) Colour map of ${\hat{\phi }}_{D}(x,y)$. The blue and green circles indicate the original defects and their mirror images about the unit circle, respectively. (*b*) Boundary values of ${\hat{\phi }}_{D}(x,y)$ and $\psi_{D}(x,y)$ along the unit circle. We computed ${\hat{\phi }}_{D}(x,y)$ from $\phi_{D}(x,y)$ by equation (2.1).

It is noted that our expression can be obtained by the method of images in electromagnetics. $\log(1-{\bar{z}}_{k}z)$ in $f_{D}(z)$ represents a complex potential by the pole at a mirror image of the original *k*th defect with respect to the unit circle ∂D. In other words, $\log(1-{\bar{z}}_{k}z)$ is the potential of a defect with the same topological charge *q*_*k*_ located at $z=1/{\bar{z}}_{k}$ (the reflection of the point *z*_*k*_ about the unit circle). Therefore, the derived potential $f_{D}(x,y)$ comprises the potentials of the original defects, log (*z* − *z*_*k*_), and those of their mirror images, $\log(1-{\bar{z}}_{k}z)$. Figure 1 shows an example of $\phi_{D}$ where *N* = 2, *q*_1_ = *q*_2_ = +1/2, *z*_1_ = 0.75e^i*π*/4^, and *z*_2_ = 0.85e^−i*π*/2^. The alignment angle is exactly equal to the tangential angle of the boundary of the unit disc (figure 1*a*) and $\phi_{D}(x,y)$ increases linearly in [0, *π*) and [*π*, 2*π*) along the unit circle (figure 1*b*).

## 3. Explicit calculation of cell alignment in non-circular geometries using conformal mappings

**Figure 2.**
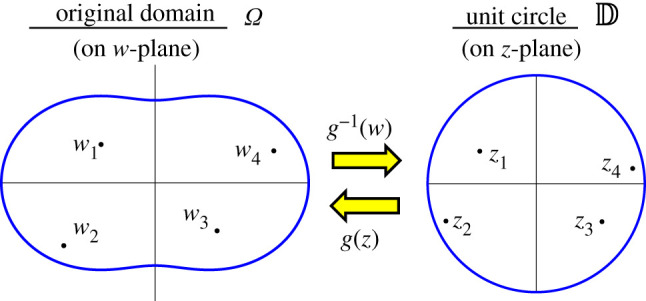
Schematic of geometries and defect positions for the calculation of cell alignment in a non-circular geometry Ω.

In this section, we extend the explicit expression (2.13) to the generalized case for a simply connected and bounded domain Ω in C which is bounded by a Jordan curve as shown in figure 2. We denote points of the domain Ω and the open unit disc D by *w* = *u* + i*v* and *z* = *x* + i*y*, respectively, which means that Ω and D are defined on the *w*-plane and the *z*-plane, respectively. We assume that there are *N* topological defects at *w*_*k*_ = *u*_*k*_ + i*v*_*k*_ with a topological charge of *q*_*k*_ for *k* = 1, …, *N*. Considering proposition 2.1 and remark 2.2, we assume the sum of the topological charge $\sum_{k=1}^{N}q_{k}$ to be equal to 1. We denote the set of defect positions in Ω by $W=\{w_{1},w_{2},\ldots ,w_{N}\}$. According to the Riemann mapping theorem, there always exists a one-to-one conformal mapping *g* that maps the open unit disc D to Ω [26]. We denote the set of topological defects $Z^{*}=\{g^{-1}(w_{1}),g^{-1}(w_{2}),\ldots ,g^{-1}(w_{n})\}\subset D$ mapped from W. Since the boundaries ∂Ω and ∂D are Jordan curves, it follows from Carathéodry’s theorem that the conformal mapping *g* extends continuously and one-to-one to the closed unit disc $\bar{D}$ [27]. Therefore, the boundary of the domain Ω is parametrically defined as the image of ∂D using *g*

3.1 $$\partial \Omega =\{w(\theta )=u(\theta )+iv(\theta )=g(e^{i\theta })\;|\;\theta \in [0,2\pi )\}.$$

The problem here is to calculate the alignment angle *ϕ*(*u*, *v*) under the condition that the cells align along the boundary ∂Ω. Using the same discussion as in §2.1, we can show that the alignment angle *ϕ*(*u*, *v*) should satisfy the Laplace equation in a subdomain of Ω which does not contain any topological defects. In addition, a topological defect in Ω is defined as a singular point of the conjugate harmonic function *ψ*(*u*, *v*) of *ϕ*(*u*, *v*) which satisfies equation (2.7). Therefore, *ϕ*(*u*, *v*) should satisfy the following three conditions:

*ϕ*(*u*, *v*) is a harmonic function on Ω∖W.
For any simply connected, bounded domain *D*_*k*_ which surrounds only the *k*th defect, *ϕ*(*u*, *v*) satisfies
  3.2 $$\oint_{\partial D_{k}}\frac{\partial \phi }{\partial s}\;ds=2\pi q_{k}.$$
On the boundary ∂Ω, the restricted value $\hat{\phi }(u,v)$ is equal to its tangential angle *ν*(*θ*):
  3.3 $$\;\hat{\;\phi }(u(\theta ),v(\theta ))=\nu (\theta )\;(\theta \in [0,2\pi ]).$$

**Remark 3.1.**

*ν*(*θ*) is expressed in terms of the conformal mapping *g* as follows. Since the boundary ∂Ω is a parametric curve (3.1), the arc-length of ∂Ω between *g*(e^i0^) and *g*(e^i*θ*^) is given by

3.4 $$s(\theta )=\int_{0}^{\theta }\left|\frac{dg(e^{i\tilde{\theta }})}{d\tilde{\theta }}\right|d\tilde{\theta },$$

which gives the following equation:

3.5 $$\frac{ds}{d\theta }=\left|\frac{dg(e^{i\theta })}{d\theta }\right|.$$

According to the theory of elementary differential geometry [28], the curvature of the curve *κ* is expressed as *κ* = d*ν*/d*s*. Therefore, *ν*(*θ*) is expressed in terms of the curvature as

3.6 $$\nu (\theta )=\nu (0)+\int_{0}^{s(\theta )}\kappa (\tilde{s}(\theta ))\;d\tilde{s}=\nu (0)+\int_{0}^{\theta }\kappa (\tilde{\theta })\left|\frac{dg(e^{i\tilde{\theta }})}{d\tilde{\theta }}\right|d\tilde{\theta }.$$

According to complex analysis [29], the curvature is calculated from the conformal mapping *g*(*z*) as

3.7 κ(θ)=1|g′(eiθ)|(Im[g′′(eiθ) ei(θ+π/2)g′(eiθ)]+1).

Let us derive an explicit expression of *ϕ*(*u*, *v*) which satisfies conditions (I)–(III). To this end, for *ν*(*θ*), we define

3.8 $$\mu (\theta )=\nu (\theta )-\theta +\frac{\pi }{2}.$$

Then, the Poisson–Schwartz integral formula gives

3.9 $$h(z)=\frac{1}{2\pi }\int_{0}^{2\pi }\mu (\theta )\frac{1+z\;e^{-i\theta }}{1-z\;e^{-i\theta }}\;d\theta ,$$

which is holomorphic in D and whose real part on ∂D coincides with *μ*(*θ*). Now, by using *h*(*z*) and the conformal mappings *g*(*z*) and *g*^−1^(*w*), an explicit expression of *ϕ*(*u*, *v*) which satisfies conditions (I)–(III) is given as follows.

**Theorem 3.2.**

*Let* $f_{\Omega }(w\;|\;W)$ *be the following holomorphic function on* Ω∖W:

3.10 $$f_{\Omega }(w\;|\;W)=f_{D}(g^{-1}(w)\;|\;Z^{*})+h(g^{-1}(w)),$$

*where* $f_{D}(g^{-1}(w)\;|\;Z^{*})$ *is defined by equation* (2.13) *for* *z* = *g*^−1^(*w*) *and* $Z=Z^{*}$. *Then, its real part*, ϕΩ(w | W)≡Re[fΩ(w | W)], *satisfies conditions* (I)–(III).

**Proof.**

We can consider that $f_{\Omega }(w\;|\;W)$ is a composite function of the conformal mapping *w* = *g*(*z*) and can be viewed as a function defined on the unit disc D. In what follows, we change variables from *w* to *z* and consider conditions (I)–(III) on the *z*-plane. To this end, we denote the real part $\phi_{\Omega }(w\;|\;W)$ by

3.11 ϕΩ(w | W)=ϕD(g−1(w) | Z∗)+Re[h(g−1(w))].

We first show that $\phi_{\Omega }(w\;|\;W)$ satisfies condition (I). Since *g*(*z*) and *g*^−1^(*w*) are holomorphic,

3.12 $$\begin{array}{rl} & \frac{\partial^{2}\phi_{\Omega }(w\;|\;W)}{\partial u^{2}}+\frac{\partial^{2}\phi_{\Omega }(w\;|\;W)}{\partial v^{2}}\\ & \;=\left({\left(\frac{\partial x}{\partial u}\right)}^{2}+{\left(\frac{\partial y}{\partial u}\right)}^{2}\right)\left(\frac{\partial^{2}\phi_{\Omega }(g(z)\;|\;W)}{\partial x^{2}}+\frac{\partial^{2}\phi_{\Omega }(g(z)\;|\;W)}{\partial y^{2}}\right)\\ & \;={\left|\frac{dg^{-1}(w)}{dw}\right|}^{2}\left(\frac{\partial^{2}\phi_{\Omega }(g(z)\;|\;W)}{\partial x^{2}}+\frac{\partial^{2}\phi_{\Omega }(g(z)\;|\;W)}{\partial y^{2}}\right). \end{array}$$

According to proposition 2.1, $\phi_{D}(z\;|\;Z^{*})$ is harmonic as a function of *x* and *y* on $D\setminus Z^{*}$. In addition, Re[*h*(*z*)] is also harmonic on D, from the Poisson–Schwartz formula. Therefore, the right-hand side of equation (3.12) is zero on $D\setminus Z^{*}$ and hence the sum of these real parts, $\phi_{\Omega }(w\;|\;W)$, is harmonic on Ω∖W, which means condition (I) holds.

For condition (II), it holds that

3.13 ∮∂Dk(∂ϕΩ(w | W)∂u du+∂ϕΩ(w | W)∂v dv)=∮∂Dk((∂ϕΩ(g(z) | W)∂x∂x∂u+∂ϕΩ(g(z) | W)∂y∂y∂u)du+(∂ϕΩ(g(z) | W)∂x∂x∂v+∂ϕΩ(g(z) | W)∂y∂y∂v)dv)=∮∂Dk(∂ϕΩ(g(z) | W)∂x(∂x∂u du+∂x∂v dv)+∂ϕΩ(g(z) | W)∂y(∂y∂u du+∂y∂v dv))=∮g−1(∂Dk)(∂ϕD(z)∂x dx+∂ϕD(z)∂y dy)+∮g−1(∂Dk)(∂ Re[h(z)]∂x dx+∂ Re[h(z)]∂y dy).

Since *g*^−1^(*w*) is a one-to-one mapping and *D*_*k*_ encloses only the *k*th defect located at *w*_*k*_, the inverse *g*^−1^(*D*_*k*_) in D encloses only the *k*th defect located at *z*_*k*_ = *g*^−1^(*w*_*k*_). Moreover, $\phi_{D}(z\;|\;Z^{*})$ satisfies condition (ii) in §2.2. Therefore, the first contour integral in the right-hand side of equation (3.13) is equal to 2*πq*_*k*_. For the second integral in the right-hand side of equation (3.13), since *h*(*z*) is holomorphic in D, its real part Re[*h*(*z*)] is harmonic in *g*^−1^(*D*_*k*_). Then, it follows from Green’s theorem that

3.14 ∮g−1(∂Dk)(∂ Re[h(z)]∂x dx+∂ Re[h(z)]∂y dy)=∬g−1(Dk)∇2 Re[h(z)] dx dy=0.

Thus, $\phi_{\Omega }(w)$ satisfies condition (II).

For condition (III), we substitute *w*(*θ*) = *g*(e^i*θ*^) into equation (3.10)

3.15 $$f_{\Omega }(w(\theta )\;|\;W)=f_{D}(e^{i\theta }\;|\;Z^{*})+h(e^{i\theta }).$$

According to proposition 2.1, ${\hat{\phi }}_{D}(\cos\theta ,\sin\theta )$ is equal to *θ* − *π*/2. In addition, Re[*h*(e^i*θ*^)] is equal to *ν*(*θ*) − *θ* + *π*/2 from the definition of *h*(*z*). Therefore, the restricted value ${\hat{\phi }}_{\Omega }(u,v\;|\;W)$ is equal to *ν*(*θ*) on the boundary ∂Ω. ▪

**Figure 3.**
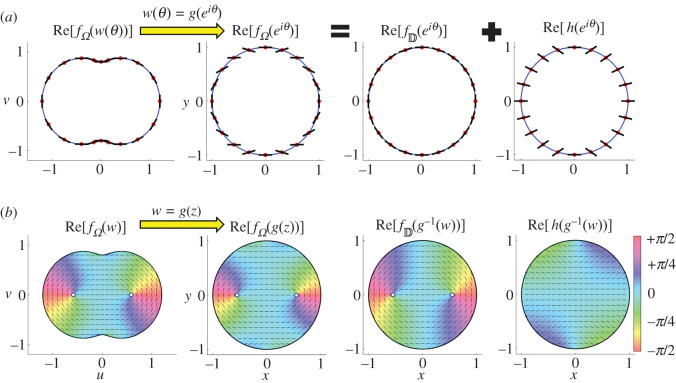
Schematic view of the calculated orientational angle in a non-circular region using conformal mapping *g*(*z*) = *z* + 0.2*z*^3^. (*a*) Boundary condition for the real part of $f_{\Omega }(w(\theta )\;|\;W)$ and its decomposition to the boundary conditions for the real parts of $f_{D}(g^{-1}(w(\theta ))\;|\;Z^{*})$ and *h*(*g*^−1^(*w*(*θ*)). (b) Real parts of the complex functions $f_{\Omega }(w\;|\;W)$, $f_{\Omega }(g^{-1}(w)\;|\;Z^{*})$, $f_{D}(g^{-1}(w)\;|\;Z^{*})$ and *h*(*g*^−1^(*w*)).

Based on theorem 3.2, we propose a method to calculate the alignment angle in a non-circular domain Ω using conformal mappings as follows. We illustrate an example when *g*(*z*) = *z* + 0.2*z*^3^ in figure 3. First, the boundary condition for $\phi_{\Omega }(w)$ is interpreted as the boundary condition for $\phi_{\Omega }(g(z))$ on D. Then, the boundary condition is decomposed to those for the real parts of $f_{D}(z\;|\;Z^{*})$ and *h*(*z*) (figure 3*a*). Since *μ*(*θ*) (i.e. the boundary condition for Re[*h*(*z*)]) represents the difference of the two boundary conditions (2.12) and (3.3), *h*(*z*) is interpreted as an additional term for satisfying the boundary condition (3.3), which is determined only by the conformal mapping *g*. From the two boundary conditions, $f_{D}(z\;|\;Z^{*})$ and *h*(*z*) are determined from equations (2.13) and (3.9), respectively. By adding these two functions and using the conformal mapping *z* = *g*^−1^(*w*), we can calculate the alignment angle at arbitrary points in Ω (figure 3*b*).

## 4. Prediction of defect positions by numerical minimization of Frank elastic energy

The alignment angle expressions in §§2.2 and 3 are functions of the defect positions *z*_*k*_ or *w*_*k*_, where those positions can be arbitrary in the domain. However, experimental studies of cell culturing have shown that defects tend to be generated on certain points which minimize the total elastic energy because cells could actively migrate [9,10]. Therefore, it is possible to predict defect positions by minimizing the elastic energy with respect to them. This section provides a systematic method for minimizing the elastic energy using formulae of complex analysis.

Since the complex potential (3.10) has singular points at defects, we should exclude the neighbourhoods of the defects in computing the elastic energy so that it does not diverge to infinity. For that purpose, we define a small disc centred at the *k*th defect $w_{k}\;(k=1,\ldots ,N)\in \Omega$ as *D*_*k*_(*ε*) = {*w*_*k*_ + *r* e^−i*θ*^| 0 ≤ *r* ≤ *ε*, 0 ≤ *θ* ≤ 2*π*} (0 < *ε* ≪ 1). Then, the elastic energy *F* is calculated as

4.1 $$F=\frac{K}{2}\int_{\tilde{\Omega }}|\nabla \phi_{\Omega }(u,v){|}^{2}\;du\;dv,$$

where $\tilde{\Omega }=\Omega \setminus \overset{N}{\underset{k=1}{⋃}}D_{k}$. We take *K*/2 = 1 because the constant *K* is not concerned with the minimization of the energy with respect to defect positions. By using the Cauchy–Riemann equations for the complex potential (3.10), the integrand in (4.1) can be expressed by complex differentiation as follows:

4.2 $$|\nabla \phi_{\Omega }{|}^{2}=|\partial_{w}f_{\Omega }(w){|}^{2}=(\partial_{w}f_{\Omega }(w))\bar{\left(\partial_{w}f_{\Omega }(w)\right)}=\bar{\partial_{w}}((\partial_{w}f_{\Omega }(w))\bar{\;f_{\Omega }(w)}),$$

where $\partial_{w}\;:\;=\partial /\partial w=\frac{1}{2}(\partial /\partial u-i(\partial /\partial v))$ and ${\bar{\partial }}_{w}\;:\;=\partial /\partial \bar{w}=\frac{1}{2}(\partial /\partial u+i(\partial /\partial v))$. Hence, applying the complex form of Green’s theorem $\iint_{\tilde{\Omega }}{\bar{\partial }}_{w}f\;(u,v)\;du\;dv=(1/2i)\oint_{\partial \tilde{\Omega }}f\;(u,v)\;dw$, the elastic energy *F* can be converted to a contour integral form:

4.3 $$F=\frac{1}{2i}\oint_{\partial \tilde{\Omega }}(\partial_{w}f_{\Omega }(w))\bar{\;f_{\Omega }(w)}\;dw.$$

Here, equation (4.3) is rewritten as a contour integral on the *z*-plane as

4.4 $$F=\frac{1}{2i}\oint_{\partial \tilde{D}}(\partial_{z}f_{\Omega }(g(z)))\bar{\;f_{\Omega }(g(z))}\;dz,$$

where $\tilde{D}=D\setminus \overset{N}{\underset{k=1}{⋃}}g^{-1}(D_{k})$ and $\partial_{z}\;:\;=\partial /\partial z=\frac{1}{2}(\partial /\partial x-i(\partial /\partial y))$. Using equation (3.10), equation (4.4) is rewritten as

4.5 $$\begin{array}{rl} F & =\frac{1}{2i}\oint_{\partial \tilde{D}}\left(\sum_{k=1}^{N}\left(-\frac{iq_{k}}{z-z_{k}}+\frac{iq_{k}{\bar{z}}_{k}}{1-{\bar{z}}_{k}z}\right)+\partial_{z}h(z)\right)\\ & \;\times \left(\sum_{k=1}^{N}(iq_{k}\log(\bar{z}-{\bar{z}}_{k})+iq_{k}\log(1-z_{k}\bar{z}))+\bar{h(z)}-\frac{\pi }{2}\right)dz, \end{array}$$

where *z*_*k*_ = *g*^−1^(*w*_*k*_). In this way, the elastic energy can be explicitly described as a function of the defect positions *z*_*k*_. In what follows, we consider the minimization of the energy *F* in $\tilde{D}$ with respect to *z*_*k*_ instead of the minimization with respect to *w*_*k*_. Once the minimizer of *F* is obtained, *w*_*k*_ is obtained as *w*_*k*_ = *g*(*z*_*k*_).

To minimize *F*, we differentiate *F* with respect to ${\bar{z}}_{k}$, giving

4.6 $$\begin{array}{rl} \frac{\partial F}{\partial {\bar{z}}_{k}} & =\frac{1}{2i}\oint_{\partial \tilde{D}}\left(\sum_{k=1}^{N}\left(\frac{iq_{k}z}{{\left(1-{\bar{z}}_{k}z\right)}^{2}}\right)\right)\left(\sum_{k=1}^{N}(iq_{k}\log(\bar{z}-{\bar{z}}_{k})+iq_{k}\log(1-z_{k}\bar{z}))+\bar{h(z)}-\frac{\pi }{2}\right)dz\\ & \;+\frac{1}{2i}\oint_{\partial \tilde{D}}\left(\sum_{k=1}^{N}\left(-\frac{iq_{k}}{z-z_{k}}+\frac{iq_{k}{\bar{z}}_{k}}{1-{\bar{z}}_{k}z}\right)+\partial_{z}h(z)\right)\left(\sum_{k=1}^{N}-\frac{iq_{k}}{\bar{z}-{\bar{z}}_{k}}\right)dz. \end{array}$$

Since $\partial F/\partial {\bar{z}}_{k}$ is considered the gradient of *F* [30], the total energy *F* can be numerically minimized by the steepest descent method as follows:

4.7 $$z_{k}^{\mathrm{new}}⟵z_{k}^{\mathrm{old}}-\alpha \frac{\partial F}{\partial {\bar{z}}_{k}},$$

where *α* is a small positive constant.

**Figure 4.**
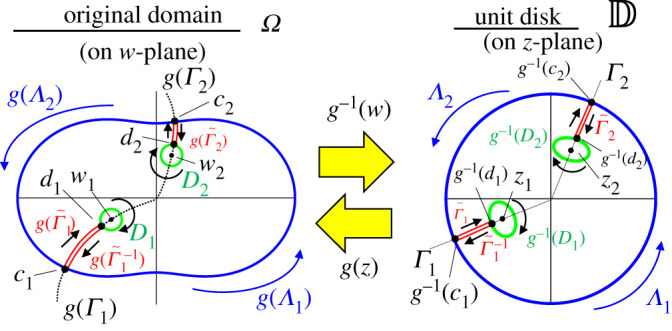
Schematic diagram of integral path on the original space Ω and the unit disc D.

To evaluate the contour integral of the elastic energy on $\tilde{D}$, we set the integral path as shown in figure 4. We define the half-line in the *z*-plane from the centre of the unit disc D through the defect position *z*_*k*_ as $\Gamma_{k}=\{t\;e^{i\;\arg(z_{k})}\;|\;t\geq 0\}$ for *k* = 1, …, *N*. Let *c*_*k*_ be an intersection point in the *w*-plane between $g(\Gamma_{k})$ and ∂Ω and *d*_*k*_ be the intersection point in the *w*-plane between *g*(Γ_*k*_) and *D*_*k*_ which satisfies |*g*^−1^(*d*_*k*_)| > |*z*_*k*_|. Then, we define a straight path in the *z*-plane from *g*^−1^(*c*_*k*_) to *g*^−1^(*d*_*k*_) as ${\tilde{\Gamma }}_{k}=\{(1-t)\;e^{i\;\arg(z_{k})}\;|\;|g^{-1}(d_{k})|\leq t\leq 1\}$ and its inverse given by ${\tilde{\Gamma }}_{k}^{-1}=\{t\;e^{i(\arg(z_{k})-2\pi )}\;|\;|g^{-1}(d_{k})|\leq t\leq 1\}$. We denote by $\Lambda_{k}$ the contour from *g*^−1^(*c*_*k*_) to *g*^−1^(*c*_*k*+1_) in ∂D. *c*_*N*+1_ is identified with *c*_1_. The contour integral was evaluated along ${\tilde{\Gamma }}_{k}$, *g*^−1^(∂*D*_*k*_) in the clockwise direction, ${\tilde{\Gamma }}_{k}^{-1}$ and $\Lambda_{k}$ in the counter-clockwise direction, for *k* = 1, 2, …, *N*.

## 5. Numerical experiments

### 5.1. Computational implementation

In all the calculations in this paper, we used Matlab 2021a. To calculate the complex logarithm log (*z* − *a*), we re-defined the log function as

5.1 $$\log(z-a)\;:\;=\log((z-a)\;e^{-i\;\arg(-a)})+i\;\arg(-a).$$

By this re-definition, we chose a branch cut of log (*z* − *a*) as {*a* + *t*exp (i arg(*a*)) | *t* > 0}.

For numerical calculation of the cell alignment and integration of the elastic energy, we divided the boundary of the unit disc ∂D into *M* = 4000 equal arcs by *M* points *ζ*_*m*_ = e^i2*πm*/*M*^ (*m* = 0, 1, …, *M* − 1). We used the trapezoidal rule in calculating the contour integral in equations (4.5) and (4.6).

To compute the value of the analytic function *h*(*z*) on $\bar{D}$ in theorem 3.2 from its known real part on the boundary, we used a Fourier expansion series as follows. Since *μ*(*θ*) is a real and periodic function of *θ* ∈ [0, 2*π*), *μ*(*θ*) is expressed as the following Fourier series expansion:

5.2 $$\mu (\theta )=\sum_{n=0}^{\infty }(a_{n}\cos n\theta +b_{n}\sin n\theta ),$$

where *a*_*n*_ and *b*_*n*_ are the Fourier series coefficients for the cosine and sine terms, respectively. By truncating the summation in equation (5.2) at the *M*/2th term, we have an approximated value of *μ*(*θ*) at *θ*_*m*_ = 2*πm*/*M*, given by

5.3 $$\mu (\theta_{m})=\sum_{n=0}^{M^{\prime }}(a_{n}\cos n\theta_{m}+b_{n}\sin n\theta_{m}),$$

where *M*^′^ = *M*/2. The holomorphic function *h*(*z*) on D can be approximately expanded as a power series of $z_{m}=r\;e^{i\theta_{m}}$ for 0 ≤ *r* < 1 by truncating the series at the *M*^′^th term

5.4 $$\begin{array}{rl} h(re^{i\theta_{m}}) & =\sum_{n=0}^{M^{\prime }}c_{n}{\left(r\;e^{i\theta_{m}}\right)}^{n}=\sum_{n=0}^{M^{\prime }}(d_{n}\cos n\theta_{m}-e_{n}\sin n\theta_{m})r^{n}\\ & \;+i\sum_{n=0}^{M^{\prime }}(d_{n}\sin n\theta_{m}+e_{n}\cos n\theta_{m})r^{n}, \end{array}$$

where *c*_*n*_ = *d*_*n*_ + i*e*_*n*_ ($d_{n},\;e_{n}\in R$). Since the limit of the real part of equation (5.4) should continuously approach *μ*(*θ*) when *r* goes to 1, we have *a*_*n*_ = *d*_*n*_ and *b*_*n*_ = −*e*_*n*_. Therefore, the value of $h(r\;e^{i\theta_{m}})$ for 0 ≤ *r* ≤ 1 can be calculated from the Fourier series coefficients of *μ*(*θ*), given by

5.5 $$h(r\;e^{i\theta })=\sum_{n=0}^{M^{\prime }}(a_{n}\cos n\theta +b_{n}\sin n\theta )r^{n}+i\sum_{n=0}^{M^{\prime }}(a_{n}\sin n\theta -b_{n}\cos n\theta )r^{n}.$$

We calculated the coefficients *a*_*n*_ and *b*_*n*_ from the fast Fourier transform (FFT) of *μ*(*θ*). For faster calculation of the obtained *h*(*z*), *h*(*z*) was approximated by an AAA algorithm [31] programmed in Chebfun [32]. We also used conformal in the Chebfun package to get the inverse of the conformal mapping.

For the contour integrals in (4.5) and (4.6), the radius of the disc *D*_*k*_ was set to *ε* = 10^−2^. Each integral path ($\Gamma_{k}$, $\Gamma_{k}^{-1}$, $\Lambda_{k}$ and ∂*D*_*k*_) was divided into 1000 equal sections. To calculate the line integral on the paths $\Gamma_{k}$ and $\Gamma_{k}^{-1}$, we multiplied by exp (*iε*_2_) and exp ( − *iε*_2_) (*ε*_2_ = 10^−10^) for the points on $\Gamma_{k}$ and $\Gamma_{k}^{-1}$, because the paths $\Gamma_{k}$ and $\Gamma_{k}^{-1}$ are on the branch cut of the complex logarithmic function defined in (5.1).

When the energy was minimized by the steepest descent method shown in (4.7), the step size *α* was set to 10^−3^ throughout the simulations. If the distance between two defects became less than 2*ε*, the update for the two defects was stopped and the two defects were removed from the calculations to prevent large oscillations of the defect positions. It is noted that this event can be viewed as annihilation of a defect pair.

### 5.2. Experimental settings

To verify the prediction of the defects by minimizing the elastic energy, we considered the following domains: (A) the unit disc D and (B) non-circular domains $\Omega_{p}$ (*p* = 2, 3, 4), whose boundaries were given by

5.6 $$\partial \Omega_{p}=\{(1+\beta \cos(p\theta ))\;e^{i\theta }\;|\;\theta \in [0,\;2\pi )\},$$

where *β* was set to 0.3 unless otherwise noted.

For these domains, we performed numerical demonstrations for the following cases.

*Case A: Unit disc.* First, we considered the simplest case of two +1/2 defects at *z*_1_ = *x*_*d*_ and *z*_2_ = −*x*_*d*_ (0 < *x*_*d*_ < 1) in the unit disc D to verify the consistency with the previous study [9] (Case A-1). The minimizer of the elastic energy with respect to *x*_*d*_ was determined by calculating the elastic energy and its derivative for various parameters ranging from 0.020 to 0.980 in steps of 0.001. Next, to show the validity of the steepest descent method (4.7), the elastic energy was minimized by the steepest descent method (4.7) (Case A-2). At the initial state, two +1/2 defects are located at *z*_1_ = 0.9e^i*π*/2^ and *z*_2_ = 0.5 in D. In addition, we considered minimization by the steepest descent method for the case of three +1/2 defects at *z*_1_, *z*_2_ and *z*_3_ and one −1/2 defect at *z*_4_ (Case A-3). In this case, we put *z*_1_ = 0.4e^i*π*/4^, *z*_2_ = 0.5e^i3*π*/4^, *z*_3_ = 0.6e^i5*π*/4^, and *z*_4_ = 0.7e^i7*π*/4^ initially.

*Case B: Non-circular domains $\Omega_{p}$.* We predicted the defect positions in non-circular domains $\Omega_{p}$ (*p* = 2, 3, 4) by the steepest descent method (4.7) (Case B-1). The initial defect positions were set as follows. For $\Omega_{2}$, two +1/2 defects were put at *z*_1_ = 0.6e^i*π*/4^ and *z*_2_ = −*z*_1_. For $\Omega_{3}$, three +1/2 defects were put at *z*_1_ = 0.8e^i*π*/9^, *z*_2_ = 0.8e^i7*π*/9^, *z*_3_ = 0.8e^i13*π*/9^ and one −1/2 defect was put at *z*_4_ = 0.01e^−i/100^. For $\Omega_{4}$, four +1/2 defects were put at *z*_1_ = 0.8e^i*π*/8^, *z*_2_ = 0.8e^i5*π*/8^, *z*_3_ = 0.8e^i9*π*/8^, *z*_4_ = 0.8e^i13*π*/8^, and one −1 defect was put at *z*_5_ = 0.01e^−i/100^. To demonstrate that there were multiple local minima of the elastic energy for the domain $\Omega_{3}$, minimization of the elastic energy was performed by the steepest descent method (4.7) for two other initial conditions: *z*_1_ = *g*^−1^(0.3), *z*_2_ = 0.95e^i2*π*/3^, $z_{3}=\bar{z_{2}}$, *z*_4_ = 0 (Case B-2) and *z*_1_ = *g*^−1^(0.4), *z*_2_ = 0.95e^i2*π*/3^, $z_{3}=\bar{z_{2}}$, *z*_4_ = 0 (Case B-3). To explain the multiple local minima, we calculated the energy landscapes for specific defect numbers and positions as follows. First, we considered two +1/2 defects in $\Omega_{3}$ and parametrized the defect positions as *z*_1_ = *r*_1_e^i2*π*/3^ and *z*_2_ = *r*_1_e^i4*π*/3^ (*r*_1_ > 0) (Case B-4). The minimizer of the elastic energy was obtained with respect to *r*_1_ from the calculated energy landscape. Then, we considered three +1/2 defects and one −1/2 defect in $\Omega_{3}$ (Case B-5). In this case, two +1/2 defects were fixed at the positions obtained in Case B-4. The other +1/2 defect and one −1/2 defect were located at *z*_3_ = *r*_2_ (*r*_2_ > 0) and *z*_4_ = 0, respectively. The elastic energy landscape was calculated by changing the value of *r*_2_. For Cases B-4 and B-5, we set the values of *β* in equation (5.6) to 0, 0.15 and 0.30.

### 5.3. Results and discussion

**Figure 5.**
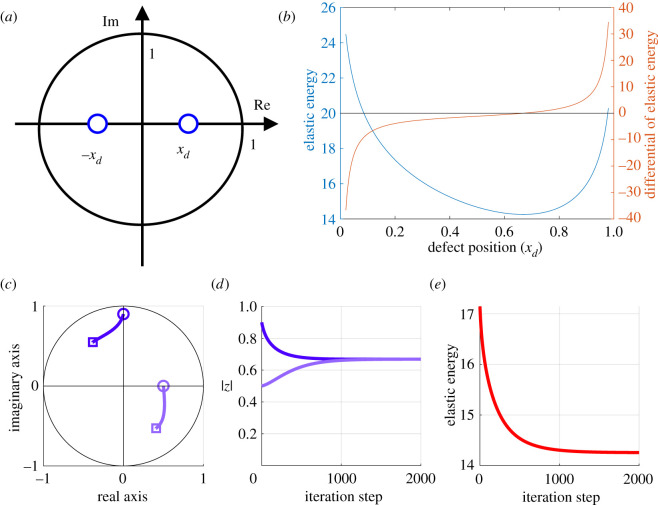
Predicted defect positions which minimize the elastic energy for two +1/2 defects in a unit disc D. (*a*,*b*) Numerical calculation of the elastic energy and its derivative in Case A-1. (*a*) Schematic of the defect positions in D for Case A-1. (*b*) Calculated elastic energy and its derivative with respect to *x*_*d*_. (*c*–*e*) Minimization of the elastic energy by the steepest descent algorithm in Case A-2. (*c*) Trajectories of the defects through the iteration of the algorithm. The circle and square markers indicate initial and final defect positions, respectively. (*d*,*e*) Temporal evolution of (*d*) the absolute number of defect positions and (*e*) the elastic energy.

Figure 5*a*,*b* shows the parametrically calculated elastic energy and its derivative in Case A-1. As shown in figure 5*b*, the elastic energy was minimized when *x*_*d*_ was 0.669, which is nearly equal to the theoretical value 5^−1/4^ reported in Duclos *et al.* [9]. We also confirmed that the theoretical value 5^−1/4^ could be re-derived using proposition 2.1 (see electronic supplementary material). A similar result was obtained in Case A-2, where the steepest descent algorithm was used for the minimization of the elastic energy (figure 5*c*–*e*). The elastic energy monotonically decreased as the algorithm proceeded, which resulted in convergence of the defect positions |*z*_1_| and |*z*_2_| to around 0.669. In addition, we confirmed that the defects were always collinearly placed in the final state, with the positions depending on the initial point of the defects located asymmetrically.

We numerically confirmed that the final defect positions depended on the initial positions because the minimization problem for the disc domain has infinitely many solutions due to the circular symmetry. Even so, the proposed method can obtain an optimized defect position because the gradient of the elastic energy vanishes once the defect positions converge to one of the minimizers.

**Figure 6.**
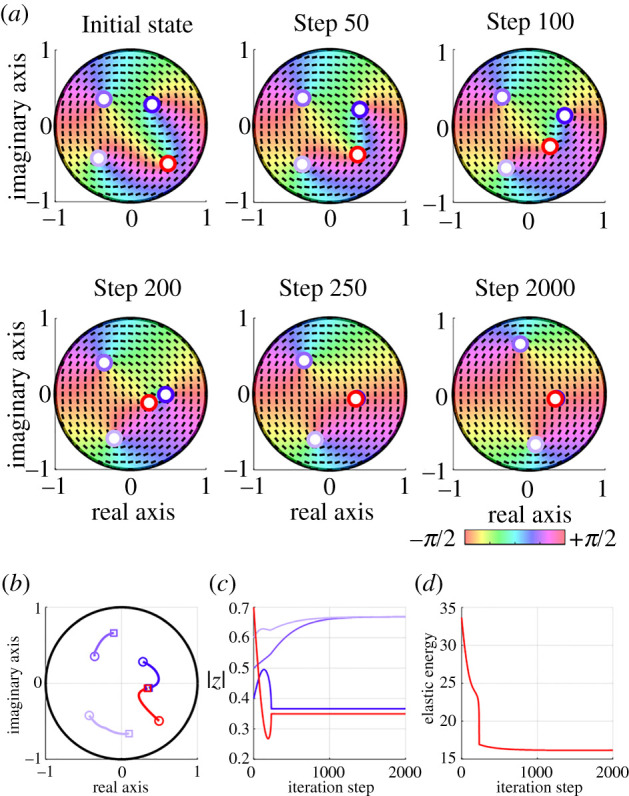
Iterative minimization of the elastic energy in Case A-3. (*a*) Spatial distribution of the orientation angle during the minimization algorithm. The bluish and reddish circles indicate +1/2 and −1/2 defects, respectively. At steps 250 and 2000, the red and blue circles almost overlap because the two defects approached each other between step 200 and step 250. (*b*) Trajectories of the defects during the algorithm. The circle and square markers indicate the initial and final defect positions, respectively. (*c*,*d*) Temporal evolution of (*c*) the absolute number of the four defects and (*d*) the elastic energy.

Figure 6 shows the iterative minimization of the elastic energy in Case A-3 where there were four defects in the unit disc D. In this case, a pair of +1/2 and −1/2 defects approached each other as the algorithm proceeded (figure 6*a*–*c*). After the attracted defects were removed, the remaining two +1/2 defects moved to the same points where the energy was a minimum as in figure 5. Throughout the algorithm, the energy monotonically decreased (figure 6*d*), which means that the minimization algorithm certainly worked even when there were initially four defects in D.

**Figure 7.**
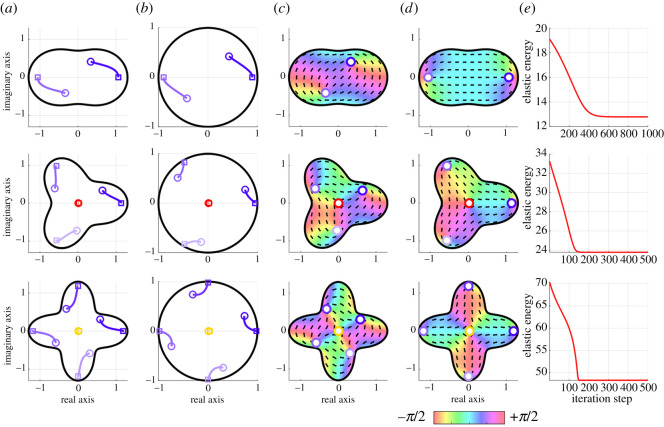
Prediction of defect positions in the various non-circular shapes $\Omega_{p}$. (Top) *p* = 2, (middle) *p* = 3, (bottom) *p* = 4. (*a*,*b*) Trajectories of defects in (*a*) the original shapes and (*b*) the unit disc mapped from the original domain. (*c*,*d*) Distribution of orientation angles at (*c*) the initial and (*d*) final states. (*e*) Time evolution of the elastic energy. The circle and square markers indicate the initial and final positions of defects, respectively. The bluish, red and yellow circles indicate +1/2, −1/2 and −1 defects, respectively.

The iterative minimization of the elastic energy also worked for the non-circular geometry case (Case B-1), as shown in figure 7. In all geometries, the elastic energy certainly decreased throughout the algorithm iterations, and the defects were finally located around the tips of lobes. It should be noted that the results for *p* = 4 were similar to one reported in the cells in a star-shaped geometry, where +1/2 defects tended to be generated around the tips of the star [10].

**Figure 8.**
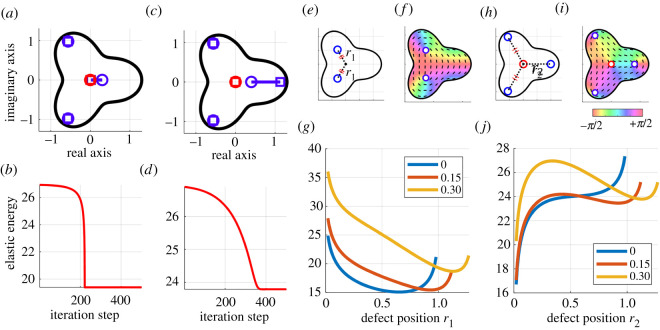
Demonstration of multiple equilibrium states due to differences in the initial defect positions. (*a*,*b*) Attracting (Case B-2) and (*c*,*d*) repelling (Case B-3) of +1/2 (bluish lines) and −1/2 (reddish lines) defects. (*a*,*c*) Trajectories of defect positions during the minimization. The circle and square markers indicate the initial and final positions of the defects, respectively. (*b*,*d*) Time evolution of the elastic energy during the minimization. (*e*–*j*) Calculation of elastic energy landscapes for (*e*–*g*) two defects (Case B-4) and (*h*–*j*) four defects (Case B-5). (*e*,*h*) Settings of the defect positions for (*e*) Case B-4 and (*h*) Case B-5. (*f*, *i*) Distribution of orientational angles corresponding to (*e*,*h*). (*g*,*j*) Relationship between the defect positions and the elastic energy. In the calculation of (*j*), the two +1/2 defects located in the left half-plane were determined from the local minima calculated in (*g*).

The equilibrium points after the iteration depended on the initial defect positions since there can be several local minima for the elastic energy function. For example, when +1/2 and −1/2 defects were closely positioned in the domain $\Omega_{3}$ (Case B-2), the two defects were attracted during the minimization (figure 8*a*,*b*). On the other hand, when the initial distance between the two defects was longer (Case B-3), the +1/2 defect moved toward the boundary of the domain. After the iteration, there remained four defects even after the minimization (figure 8*c*,*d*). Therefore, there are at least two equilibrium states in this shape. To further investigate the dependence of the initial defect positions on the final states for these cases, we calculated the elastic energy landscapes for Cases (B-2) and (B-3). For two defects as shown in figure 8*e*,*f* (Case B-4), there was only one minimal point of the elastic energy (figure 8*g*). When one +1/2 and one −1/2 point were added as shown in figure 8*h*,*i* (Case B-5), there were one local maxima and two local minimal points at *r*_1_ = 0 and *r*_1_ > 0 for *β* = 0.15, 0.30 (figure 8*j*). These analyses support there being two equilibrium states shown in figure 8*a*,*d* due to the existence of multiple local minima and that the proposed method will predict which state is the final state from the initial defect positions.

The proposed method allowed the calculation of cell alignment in non-circular shapes as shown in figures 3 and 7. Although we assumed a smooth boundary in the calculation of the cell alignment in §3, it is expected that the proposed method can also be applied to piecewise smooth shapes such as polygons by using Schwarz–Christoffel mappings. However, since such non-smooth shapes have singularities at corners, the elastic energy would diverge to infinity due to the singularities in the conformal mappings. In future work, we will examine solutions for such problems and investigate the effects of the corner singularities on the prediction of the defect positions.

The proposed method also enabled direct differentiation of the elastic energy with respect to the defect positions and the minimization of the elastic energy. This calculation was possible because the constructed analytic function *h*(*z*) does not depend on the defect positions. Moreover, the integration of the elastic energy is in contour integral forms, which makes it easier to calculate the integral around the singular point *z*_*k*_ compared with surface integral forms. These advantages of the proposed method contribute to the calculation of the defect positions in non-circular domains as shown in figures 6 and 7. Especially, the result for the four-leaves domain (figure 7) is consistent with the previous result which can control positions of +1/2 defects [10]. Therefore, the proposed method could be applied to predicting the defect positions and design of geometries for controlling defect positions. Our numerical results predicted the exact minimal point for various geometries, but the results have not been experimentally confirmed. Therefore, future work will include cell culturing experiments to verify the proposed calculation method.

## 6. Conclusion

This study proposed a systematic method for calculating the alignment angle and predicting defect positions which minimize the elastic energy in cell populations at a confluent state. Using several complex analysis techniques, we derived an explicit expression of the cell alignment angle in a unit disc in terms of the defect positions. The derived expression was extended to an angle calculation for non-circular geometries by using conformal mapping. By taking advantage of the explicit expression of the alignment angle in terms of the defect positions, we proposed a method for calculating the elastic energy and its derivative with respect to defect positions and minimizing the elastic energy by a steepest descent method. The validity of the methods was confirmed by numerical simulations. The results for the unit disc were consistent with the previous study, [9] while qualitative agreement with the previous study [10] was also confirmed for non-circular geometries with four lobes. Therefore, the proposed method will be a powerful tool for designing geometries to control defect positions.

## Data accessibility

The program codes are available in the electronic supplementary material [33].
